# Thymoma Patients Treated in a Phase I Clinic at MD Anderson Cancer Center: Responses to mTOR Inhibitors and Molecular Analyses

**DOI:** 10.18632/oncotarget.1015

**Published:** 2013-06-10

**Authors:** Jennifer Wheler, David Hong, Stephen G. Swisher, Gerald Falchook, Apostolia M. Tsimberidou, Thorunn Helgason, Aung Naing, Bettzy Stephen, Filip Janku, Philip J. Stephens, Roman Yelensky, Razelle Kurzrock

**Affiliations:** ^1^ Department of Investigational Cancer Therapeutics – a Phase I Clinical Trials Program, The University of Texas MD Anderson Cancer Center; ^2^ Thoracic and Cardiovascular Surgery, The University of Texas MD Anderson Cancer Center; ^3^ Foundation Medicine, University of California, San Diego; ^4^ Moores Cancer Center, University of California, San Diego

**Keywords:** advanced thymoma, mTOR inhibitors, response, targeted therapy, thymic carcinoma

## Abstract

**BACKGROUND:**

Thymomas and thymic carcinoma are rare tumors with no approved therapies. Our purpose was to analyze the molecular features and outcomes of patients referred to the Clinical Center for Targeted Therapy (Phase I Clinic).

**METHODS:**

We retrospectively reviewed the medical records of consecutive referred patients with advanced/metastatic thymoma or thymic carcinoma

**RESULTS:**

Twenty-one patients were identified (median age 52 years; 10 women; median number of prior systemic therapies = 2). Six of 10 patients (60%) treated with mTOR inhibitor combination regimens achieved stable disease (SD) ≥12 months or a partial response (PR). For patients treated on mTOR inhibitor regimens (N = 10), median time to treatment failure (TTF) was 11.6 months versus 2.3 months on last conventional regimen prior to referral (p=0.024). Molecular analyses (performed by next generation sequencing in seven patients and single polymerase chain reaction (PCR)-based assays in an additional six patients) showed diverse actionable mutations: *PIK3CA* (1 of 12 tested; 8%); *EGFR* (1 of 13; 8%); *RET* (1 of 7; 14%); and *AKT1* (1 of 7; 14%). Of two patients with *PIK3CA* or *AKT1* mutations, one was treated with an mTOR inhibitor-based regimen and achieved 26% regression with a TTF of 17 months.

**CONCLUSION:**

Patients with advanced/metastatic thymoma or thymic carcinoma demonstrated prolonged TTF on mTOR inhibitor-based therapy as compared to prior conventional treatment. Heterogeneity in actionable molecular aberrations was observed, suggesting that multi-assay molecular profiling and individualizing treatment merits investigation.

## INTRODUCTION

Thymic epithelial tumors include thymomas and thymic carcinomas. Though rare (0.2-1.5% of all malignancies)[1], they represent the most common tumor of the anterior mediastinum[2]. Thymomas account for approximately 20 percent of mediastinal tumors and, in advanced stages are typically aggressive. Thymic carcinomas are malignant epithelial tumors without the thymus-like features of thymomas[3]. The molecular pathogenesis of these tumors remains poorly elucidated, although retrospective case studies have demonstrated the presence of rare molecular aberrations in important oncogenes (*EGFR, HER2, KIT, KRAS*, and *BCL2*), and tumor suppressor genes (*TP53*, and *CDKN2A/B*)[4-9].

Surgical resection is the mainstay of treatment for early stage disease[10]. Advanced/metastatic disease is treated with multimodality therapy including induction chemotherapy, surgery and radiation/chemotherapy[5]. Combination chemotherapy regimens used most often, either alone or as part of multimodality therapy, include cisplatin, doxorubicin and cyclophosphamide (PAC) with or without prednisone, cisplatin, doxorubicin, cyclophosphamide and vincristine (ADOC), etoposide and cisplatin (EP), and, etoposide, cisplatin and ifosfamide (VIP)[10]. Anecdotal responses to mTOR inhibitors have been reported[11, 12]. Herein, we describe the clinical and molecular characteristics and outcomes of 21 patients with advanced/metastatic thymoma or thymic carcinoma referred to the Clinical Center for Targeted Therapy (Phase I Clinic).

## RESULTS

### Patient Characteristics

A total of 21 consecutive patients with advanced/metastatic thymoma or thymic carcinoma were referred to the Clinical Center for Targeted Therapy (Phase I Clinic). Their median age was 52 years (range, 26-73 years) (Table 1). Ten patients (48%) were women. The median number of prior therapies in the metastatic setting was 2 (range, 0-6). The most common metastatic sites were lung, pleura, and lymph nodes.

**Table 1:** Characteristics of 21 patients with advanced/metastatic thymoma or thymic carcinoma

Characteristics	Group	Number of patients (n=21)	%
Age	≤60	14	66.7
	>60	7	33.3
Sex	Women	10	47.6
	Men	11	52.4
History of thromboembolism	No	13	61.9
	Yes	8	38.1
Number of prior therapies	<3	14	66.7
	≥3	7	33.3
ECOG performance status	<1	9	42.9
	≥1	12	57.1
Number of metastatic sites	≤2	5	23.8
	>2	16	76.2
History of surgery	No	9	42.9
	Yes	12	57.1
History of radiation	No	7	33.3
	Yes	14	66.7
Hemoglobin (g/dL)	<11	4	19.0
	≥11	17	81.0
Platelets (K/UL)	≤440	20	95.2
	>440	1	4.8
Albumin (g/dL)	<3.5	1	4.8
	≥3.5	20	95.2
LDH (IU/L)*	≤618	19	90.5
	>618	2	9.5

Abbreviations: ECOG, Eastern Cooperative Oncology Group; LDH, lactate dehydrogenase *LDH, 618 IU/L is reported as upper limit of normal in our institution

### Molecular analyses

Of 12 patients assessed for a *PIK3CA* mutation (7 by NGS; 6 by single gene PCR sequencing, including one of whom was also assessed by NGS), one patient (8%; case #16, Table 2) had a *PIK3CA* mutation (S553T) in exon 9. One of the 13 patients (8%) assessed for an *EGFR* mutation (7 by NGS; 8 by single gene PCR-based sequencing including 2 who also had NGS) had a mutation (case #4, Table 2; T785I in exon 20) in the sample obtained five years prior to referral. Of interest no *EGFR* mutation was found by NGS in the sample obtained from a separate site, one year after referral. Twelve patients assessed for a *KRAS* mutation, 9 for an *NRAS* mutation, and 9 for a *BRAF* mutation, were all wild-type. None of the 6 patients (cases #3, 4, 12, 14, 15, and 16, Table 2) evaluated for expression of PTEN by IHC had PTEN loss and none of the 7 patients evaluated by NGS showed PTEN abnormalities (total=11 patients evaluated for PTEN since two had both NGS and PTEN by IHC).

**Table 2:** Molecular analyses and outcome in 21 patients with advanced/metastatic thymoma or thymic carcinoma

Case No.	Diagnosis	Phase I protocol drugs (mechanism)	Best response (Recist %)a	TTF (m)b	NGS at Foundation Medicinec			PCR-based single gene assessmentd
					Somatic mutations	Amplifications	Deletions	
1	Invasive thymoma	interleukin-6 and VEGFinhibitor	SD (10.2)	4				
2	Invasive thymoma	TRAIL receptor-2 agonist	SD (−19.0)	25	none	none	none	
3	Thymic carcinoma	anthracycline, monoclonal antibody, mTOR inhibitor	SD (−17.0)	2				*EGFR*, *KRAS*, *NRAS*, *BRAF*, c-KIT: wild-type; ER, PR, Her2: negative; PTEN present by IHC‡
4	Thymic carcinoma	anthracycline, monoclonal antibody, mTOR inhibitor	SD (−24.0)	12	APC_c.4606G> T_p.E1536*(0.26,426), TP53_c.844C> T_p.R282W(0.92,129)	MCL1_gain (12,MCL1_target_1-3)	none	*EGFR*: T785I (exon 20); *KRAS*: wild-type; PTEN present by IHC‡
5	Invasive thymoma	hypomethylator (cytidineanalog)	PR (−42.0)	5				
6	Thymic carcinoma	anthracycline, monoclonal antibody, mTOR inhibitor	SD (−21.0)	18+				*PIK3CA*, *EGFR*, *KRAS*: wild-type; PDGFR- negative
7	Thymic carcinoma	farnesyltransferase inhibitor+ RAF kinase/ VEGFR inhibitor	SD (17.0)	2				
8	Thymic carcinoma	antimitotic, mTOR inhibitor	SD (−9.0)	12+	RET_c.2302C> G_p.E768Q(.05,786)	none	CDKN2A_loss (0,CDKN2A_target_1-6);CDKN2B_loss (0,CDKN2B_target_1-4)	*KRAS*: wild-type
9	Thymic carcinoma	microtubule inhibitor	PD** (20.0)	2				
10	Thymic carcinoma	histone deacetylase inhibitor, immunomodulator	PD* (20.0)	2				
11	Thymic carcinoma	microtubule inhibitor	PR (−35.0)	7	none	MCL1_gain (10,MCL1_target_1-5)	none	
12	Thymic carcinoma	anthracycline, monoclonal antibody, mTOR inhibitor	SD (−7.0)	14	none	none	none	*PIK3CA*, *EGFR*, *KRAS*, *NRAS*, *BRAF*, c-KIT: wild-type; PTEN present by IHC‡
13	Thymic carcinoma	-	-	-				*PIK3CA*, *EGFR*: wild-type
14	Thymic carcinoma	inhibitor of MEK1/MEK2 activation and kinase activity, EGFR inhibitor	too early for assessment	0+				*PIK3CA*, *EGFR*, *KRAS*, *NRAS*, *BRAF*, c-KIT, TP53, GNAQ: wild-type; ALK-1: negative; PTEN present by IHC‡
15	Thymic carcinoma	immunomodulator, mTOR inhibitor	PD* (20.0)	1				*PIK3CA*, *EGFR*, *KRAS*, GNAQ: wild-type; ER/PR/ALK-1: negative; PTEN present by IHC‡
16	Thymic carcinoma	c-Met kinase inhibitor	SD (19.0)	3+				*PIK3CA*: p.S553T (exon 9); *EGFR*, *KRAS*, c-KIT, GNAQ: wild-type; PTEN present by IHC‡
17	Invasive thymoma	anthracycline, monoclonal antibody, mTOR inhibitor	SD (−26.0)	17	AKT1_c.49G> A_p.E17K(0.43,743)	none	none	
18	Thymic carcinoma	anthracycline, monoclonal antibody, mTOR inhibitor	PD* (20.0)	4				
19	Thymic carcinoma	anthracycline, monoclonal antibody, mTOR inhibitor	PR (−30.0)	12+				
20	Invasive thymoma	nucleoside analog,Src inhibitor	SD (−16.0)	10+	none	none	none	
21	Thymic carcinoma	mTOR inhibitor, EGFR inhibitor	SD (−7.0)	3				

Abbreviations: APC, adenomatous polyposis coli; ALK, anaplastic lymphoma kinase; *BRAF*, v-Raf murine sarcoma viral oncogene homolog B1; *EGFR*, epidermal growth factor receptor; ER, estrogen receptor; GNAQ, guanine nucleotide binding protein (G protein), q polypeptide; Her2, human EGF receptor 2; IHC, immunohistochemistry; *KRAS*, V-Ki-ras2 Kirsten rat sarcoma viral oncogene homolog; mTOR, mammalian target of rapamycin; NGS, next generation sequencing; MEK, mitogen-activated protein kinase; MCL1, myeloid cell leukemia sequence 1; *NRAS*, Neuroblastoma RAS viral oncogene homolog; PR, partial response; PTEN, Phosphatase and tensin homolog; *PIK3CA*, phosphatidylinositol 3-kinase, catalytic, alpha polypeptide; PDGFR, platelet-derived growth factor receptor; PR, progesterone receptor; PD, progressive disease; RET, rearranged during transfection; SD, stable disease; TTF, time to treatment failure; TP53, tumor protein p53; VEGF, vascular endothelial growth factor

a”*” = clinical progression; “**” = new metastasis

b”+” = did not progress at the time of analysis

c includes results of genes of known relevance to cancer

d”‡” = the presence of PTEN by IHC denotes a result ‘negative' for aberration

NGS analyses were performed on tissues obtained from seven patients. One patient (case #4, Table 2) had an *APC* mutation (E1536), *TP53* mutation (R282W) and *MCL1* amplification. A second patient (case #8, Table 2) had a *RET* mutation (E768Q) and *CDKN2A/B* deletion. *MCL1* amplification was identified in a third patient (case #11, Table 2) and an *AKT1* mutation (E17K) in a fourth patient (case #17, Table 2). No molecular aberrations by NGS analyses were noted in three other patients (cases #2, 12 and 20, Table 2).

### Treatment

The median number of phase I trials per patient was one (range, 1-6). For patients treated on more than one phase I trial, we report the data from the phase I trial with the best response. Twenty patients were treated on 13 different phase I clinical trials (Table 2 and Supplemental Table 1). Most patients (n=17, 81%) received treatment with regimens that included at least one targeted agent, including 10 patients who received treatment with mTOR inhibitor combination therapies.

### Response to treatment

**Figure 1:**
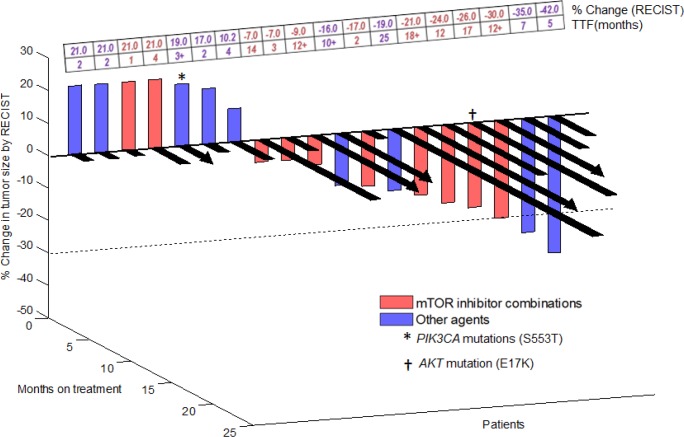
3-D Waterfall plot Best response by RECIST of 19 patients with advanced/metastatic thymoma or thymic carcinoma (one patient was not enrolled on a trial and one patient was too early for response assessment). Time-to-treatment failure (TTF) in months is represented by solid black lines and the arrow indicates that the patient was still on study when the data was censored. Patients with clinical progression or with new lesions were graphed as 20% progression. Dotted horizontal line at -30% indicates border for partial response. A comprehensive list of molecular alterations is found in Table 2.

Of 21 patients on this study, 19 were evaluable for response (Table 2; Figure 1). Two patients were not evaluable for response. One of these patients had not yet been restaged at the time of data analysis and the other patient was not enrolled on a trial (cases #14 and 13 respectively; Table 2). Of the 19 patients evaluable for response, nine patients (47%) attained either a PR (n=3; cases #5, 11 and 19; Table 2) or SD ≥12 months (n=6; cases #2, 4, 6, 8, 12, and 17; Table 2). Four patients came off study prior to post-treatment imaging evaluation due to clinical progression (all of whom were arbitrarily graphed as 20% progression in Figure 1). Six of 10 patients (60%; cases #4, 6, 8, 12, 17, and 19; Table 2) treated with an mTOR inhibitor containing regimen achieved SD ≥12 months/PR. Three of nine evaluable patients (33%; cases #2, 5, and 11, Table 2) treated with other agents achieved SD ≥12 months/PR. TTF of ≥12 months was achieved by six of 10 patients on an mTOR inhibitor-containing regimen (cases #4, 6, 8, 12, 17, and 19; Table 2; TTF = 12, 18+, 12+, 14, 17, and 12+ months, respectively) versus one of 10 patients treated with other agents (case #2; Table 2; TTF = 25 months) (p = 0.057). One of two patients (cases #16 and 17, Table 2) with a mutation in the PI3K/AKT/mTOR axis was treated with an mTOR-based regimen and achieved 26% regression with a TTF of 17 months (case #17, Table 2).

### TTF on phase I protocol compared to TTF on prior therapy

**Figure 2:**
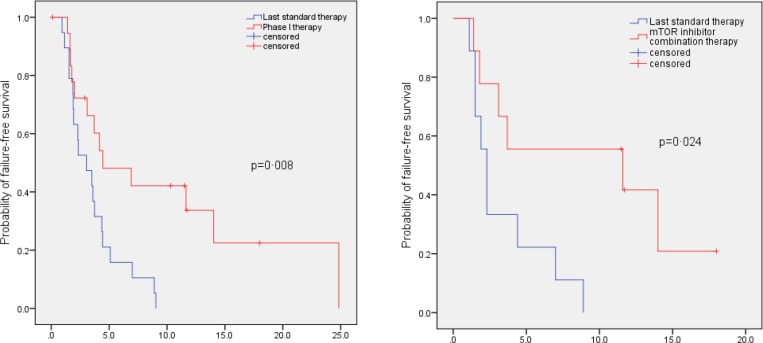
Kaplan - Meier curve to compare TTF in patients with advanced/metastatic thymoma or thymic carcinoma on their best phase I clinical trial versus TTF on their last conventional therapy before referral to the phase I clinic Tick marks represent patients still continuing on treatment and hence censored at last follow up. Panel A. Comparison of TTF in 19 patients (median of 4.5 months in phase I program vs. median of 3.0 months on their last conventional therapy before referral to phase I; p=0.008). Two patients were not included in this paired analysis as one patient did not receive a phase I trial (case #13, Table 2) and a second patient did not receive prior systemic therapy for advanced cancer (case #17, Table 2). Panel B. Comparison of TTF in nine patients treated on mTOR inhibitor combination therapies (median of 11.6 months on mTOR inhibitor combinations in phase I clinic vs. median of 2.3 months on last conventional therapy; p=0.024).

We analyzed the median TTF on a phase I study versus median TTF for the therapy immediately preceding referral to the phase I clinic. Two patients were not included in the paired analysis as one patient did not receive a phase I trial (case #13, Table 2) and a second patient did not receive prior systemic therapy for advanced cancer (case #17, Table 2). The median TTF was significantly longer on a phase I trial (4.5 months, 95% CI, 0.3–8.7 months) compared to median TTF on the last therapy before referral to phase I (3.0 months, 95% CI, 1.3–4.7 months; p=0.008; Figure 2A). We also analyzed the median TTF in a sub-group of patients on mTOR inhibitor-based combinations. The median TTF was significantly longer in nine patients (one patient [case #^17^; Table 2] was not included in the paired analysis as the patient did not receive prior systemic therapy for advanced cancer) treated on mTOR inhibitor combinations (11.6 months, 95% CI, 0.0–30.9 months) compared to median TTF on their last standard therapy prior to referral to the phase I clinic (2.3 months, 95% CI, 1.7–2.9 months; p=0.024; Figure 2B).

### Overall Survival

The median follow-up duration of surviving patients from the date of presentation to the phase I clinic was 21.4 months (range, 9.6 – 59.5 months). The median OS from the time of diagnosis of advanced/metastatic thymoma or thymic carcinoma to death or last follow up was 85.7 months (95% CI, 40.8 – 130.6 months). The median OS from the date of presentation to the phase I program was 29.2 months (95% CI, 18.7-39.7 months). At the time of analysis, 15 patients were dead.

## DISCUSSION

Advanced/metastatic thymomas and thymic carcinoma exhibit aggressive behavior[4, 13]. They are a distinct clinical entity associated with worse prognosis despite multimodal approach[14], which underscores the urgent need for the development of novel therapeutic approaches. The purpose of this study was to systematically analyze the clinical outcomes of 21 patients with advanced/metastatic thymoma and thymic carcinoma referred to a phase I clinic.

In this analysis, we observed that 9 of 19 patients (47%) evaluable for response achieved either SD ≥12 months (n=6) or a PR (n=3) (Table 2; Figure 1). This includes six of 10 patients (60%) treated on mTOR inhibitor combination therapies. TTF ≥12 months was achieved by six of 10 patients on an mTOR inhibitor-containing regimen versus one of 10 patients treated with other agents (p=0.057). Patients treated on mTOR inhibitor-containing phase I regimens also demonstrated a significantly longer TTF (median=11.6 months) compared to the TTF on the therapy they received prior to referral to phase I (median=2.3 months) (p=0.024; Figure 2B). Prior anecdotal responses to mTOR inhibitors have previously been reported as well[11, 12]

Though several signaling pathways have been explored in thymic tumors, clinical trials with EGFR, KIT, VEGF, and IGF-1R, histone deacetylase, DNA methyltransferase, tropomyosin receptor kinase A, and, cyclin-dependent kinase inhibitors documented only modest clinical responses in advanced disease[4, 5, 15]. Our results show that thymic tumors have diverse molecular abnormalities. For instance, one of 12 patients (8%) harbored a *PIK3CA* mutation; one of 13 (8%), an *EGFR* mutation; one of seven (14%), a *RET* mutation; and one of seven (14%), an *AKT1* mutation. While each of these aberrations is potentially actionable, it is apparent that treating all patients with any one targeted agent would likely be successful in only a very small subset of patients, at best. These observations underscore the need for investigating rational multiplex molecular diagnostics paired with matched targeted therapy [16].

In our analysis of patients treated on targeted therapies, however, six of 10 individuals (60%) who received mTOR inhibitor combination therapies attained either SD ≥12 months (n=5) or a PR (n=1). Results of molecular analyses were available for five of the six patients including four who had NGS analysis. One of the five patients (case #17, Table 2) had an *AKT1* mutation, and achieved tumor regression of 26% that lasted 17 months. The underlying basis of response of the other patients is unclear. It may be that some of the aberrations observed result in crosstalk with the PI3K/AKT/mTOR pathway. Alternatively, additional transcriptome or proteosome analysis might reveal aberrations or signatures indicative of PI3K/AKT/mTOR pathway activation.

Three of the nine evaluable patients treated with agents that did not include an mTOR inhibitor achieved SD ≥12 months/PR. Of interest, one patient (case #2; Table 2) treated with a TRAIL receptor-2 agonist, attained SD for 25 months versus TTF of four months on last therapy before referral to the phase I clinic. NGS analysis performed on this patient's tissue did not identify any molecular aberrations. A planned in-depth analysis of RNA/proteomics may help to understand the prolonged stable disease observed in this patient. Two other patients (cases #5 and 11, Table 2) achieved a PR, albeit of shorter duration (5 and 7 months), on decitabine (hypomethylating agent) and patupilone (microtubule inhibitor), respectively.

One patient (case #4, Table 2) showed an EGFR mutation in sample tissue obtained five years prior to referral. NGS performed on tissue obtained one year post referral failed to discern this mutation. These observations confirm the complexity of tumor heterogeneity[17]. Other aberrations included mutations in *APC*, a tumor suppressor gene often altered in colorectal cancer[18], or in *p53*[4, 19]. MCL1 amplification implicated in antiapoptic activity[20], and loss of *CDKN2A/B*, a gene that encodes inhibition of cyclin-dependent kinases[9, 21] were also seen (Table 2).

This study has several important limitations. The small sample size and retrospective nature of the analysis precludes robust statistical evaluation. In this context, the study should be considered hypothesis-generating. The responses with mTOR-based regimens are of potential interest, but could have been confounded by the use of combination therapy. In this regard, anthracyclines are known to be active in thymic cancer[4, 22]. However, three of the five patients with TTF ≥12 months who were treated with a regimen that contained both an mTOR inhibitor and an anthracycline had received a prior anthracycline containing regimen.

In conclusion, advances in the treatment of advanced/metastatic thymoma and thymic carcinoma are hampered by the rarity of these conditions. No drugs are approved for these tumors. Our study showed that six of 10 patients (60%) treated on mTOR inhibitor-containing regimen attained a SD ≥12 months/PR. The TTF on mTOR inhibitor combination therapies was significantly longer than the TTF on the last conventional therapy prior to referral to the phase I clinic (median of 11.6 vs. 2.3 months; p=0.024; Figure 2B). Diverse actionable molecular aberrations were seen in our patients including, but not limited to, mutations in *PIK3CA* (1 of 12 tested; 8%); *EGFR* (1 of 13; 8%); *RET* (1 of 7; 14%); and *AKT1* (1 of 7; 14%). Other aberrations were also observed: *TP53* mutation, *APC* mutation, *MCL1* amplification, and, *CDK2A/B* deletion. These observations highlight the heterogeneity between patients, and suggest that treating unselected patients with any one regimen is unlikely to achieve high partial or complete remission rates. Furthermore, two patients tested by NGS each had several abnormalities, making tailored treatment more complex. Of interest, three of seven patients tested by NGS showed no abnormality; further evaluation of this disease using transcriptome and/or proteomic analysis may therefore be necessary in order to fully understand its underlying biology.

## METHODS

We reviewed the electronic medical records of consecutive patients with advanced/metastatic thymoma or thymic carcinoma referred to the Department of Investigational Cancer Therapeutics (Phase I Clinical Trials Program) at The University of Texas MD Anderson Cancer Center beginning January 1, 2006. This study and all treatments were carried out in accordance with the guidelines of the MD Anderson Cancer Center Institutional Review Board.

Treatment was determined after clinical, pathologic and laboratory data were reviewed. Phase I clinical trials available for patient enrollment varied over time depending upon protocol availability at the time of presentation to the phase I clinic (Clinical Center for Targeted Therapy). Assessments, including history, physical examination, and laboratory evaluations, were done as specified in each protocol, typically before the initiation of therapy, weekly during the first cycle, and then, at a minimum, at the beginning of each new treatment cycle. Efficacy was assessed from computed tomography (CT) scans and/or magnetic resonance imaging (MRI) at baseline before treatment initiation and then about every two cycles (six to eight weeks). All radiographs were read in the Department of Radiology at MD Anderson and reviewed in the Department of Investigational Cancer Therapeutics tumor measurement clinic.

### Molecular assays

All histologies were centrally reviewed at MD Anderson Cancer Center. Molecular testing was dependent on availability of appropriately processed tissue; furthermore, assay availability evolved with time.

Molecular testing polymerase chain reaction (PCR)-based DNA sequencing (*EGFR, NRAS, KRAS, BRAF* and *PIK3CA*) and immunohistochemistry (IHC) for PTEN (DAKO antibody, Carpinteria, CA) were performed in the clinical laboratory improvement amendment (CLIA)-certified Molecular Diagnostic Laboratory at MD Anderson as described in previous publications[23-26]

Next generation sequencing (NGS) was performed at Foundation Medicine (Cambridge, MA) in seven patients with available tissue. Genomic libraries were captured for 3230 exons in 182 cancer-related genes plus 37 introns from 14 genes often rearranged in cancer and sequenced to average median depth of 734X with 99% of bases covered >100X.

### End Points and Statistical Methods

Descriptive statistics were used to summarize the baseline patients' characteristics. The Fisher's exact test was used to assess the association between categorical variables. Responses were categorized per RECIST 1.0 criteria[27] and were reported as best response. 3D-waterfall plot was used to illustrate the responses and their duration in these patients as previously described[28]. Overall survival (OS) was measured from the date of presentation to the phase I clinical trials program at MD Anderson until death from any cause or last follow-up. Patients still alive were censored for survival at the time of their last follow-up. Time-to-treatment failure (TTF) was defined as the time interval between the start of therapy and the date of disease progression or death or removal from study for any reason, whichever occurred first. Patients alive and without disease progression were censored at the last follow-up date. For patients treated on more than one phase I clinical trial, data from the best response phase I clinical trial was used for analysis. The Kaplan-Meier method [29] was used to estimate the probabilities of OS and TTF, and log-rank tests [30] were utilized to compare subgroups of patients. All tests were 2-sided, and *P* <0.05 was considered statistically significant. All statistical analyses were carried out using SPSS (version 19.0; SPSS, Chicago, IL, USA).

## SUPPLEMENTARY TABLES


