# Supplementary material for: Thymoma Patients Treated in a Phase I Clinic at MD Anderson Cancer Center: Responses to mTOR Inhibitors and Molecular Analyses
Source: Oncotarget. 2013 Jun 10;4(6):890–8. doi: 10.18632/oncotarget.1015 (PMC3757246; doi:10.18632/oncotarget.1015)
Supplement: Supplementary file 1 [file oncotarget-04-890-s001.doc]

**Thymoma Patients Treated in a Phase I Clinic at MD Anderson Cancer Center: Responses to mTOR Inhibitors and Molecular Analyses – Wheler et al**

**Supplemental Table 1**: List of clinical trials*.

| Clinical trial registry number |
| --- |
| NCT00214838 |
| NCT00761644 |
| NCT00089089 |
| NCT00244972 |
| NCT01054313 |
| NCT00420615 |
| NCT00495872 |
| NCT01192165 |
| NCT01183663 |
| NCT01110083 |
| NCT00429234 |
| NCT00940381 |

*clinical trial for AMG-655, a TRAIL receptor-2 agonist, was not registered
